# Prevalence of bronchiectasis in inflammatory bowel disease: a systematic review and meta-analysis

**DOI:** 10.3389/fmed.2024.1447716

**Published:** 2024-11-21

**Authors:** Yu Ma, Zhihui Qiang, Miaomiao Zhou, Tianyi Zhang, Zhuoyang Li, Haicheng Zhong, Yue Chang, Zimeng Ning, Yun Liu

**Affiliations:** Department of Respiratory and Critical Care Medicine, Xi’an Jiaotong University Second Affiliated Hospital, Xi’an, China

**Keywords:** inflammatory bowel disease, Crohn’s disease, ulcerative colitis, bronchiectasis, prevalence, HRCT

## Abstract

**Objective:**

The aim of this study was to conduct a systematic review and meta-analysis of the incidence of inflammatory bowel disease-associated bronchiectasis (IBD-BE) and to explore the possible risk factors for IBD-BE, which could help to understand the pulmonary involvement in patients with IBD and to determine the global incidence of the disease.

**Methods:**

We searched PubMed and EMBASE databases to identify information on the prevalence of IBD-BE among IBD patients in the published literature. Information was extracted on study design, country, year, IBD-BE testing method, IBD characteristics, number of IBD-BE cases and total number of IBD patients, and factors associated with IBD-BE. We conducted meta-analyses using random-effects or fixed-effects models to estimate the prevalence of IBD-BE among IBD patients.

**Results:**

Out of a total of 682 studies, we identified 16 studies that reported prevalence. These studies used a heterogeneous approach to identify IBD-BE. In these 16 studies, there were 92,191 patients with IBD, of whom 372 cases of IBD-BE were identified. The results of the meta-analysis showed that the overall prevalence of IBD-BE in IBD derived from the use of a random effects model was 5.0% (95% CI 2.0–12.0%). In contrast, the prevalence of IBD-BE in studies using high-resolution chest computed tomography (HRCT) imaging was 12% (95% CI 4–39%) using a random-effects model. When only retrospective studies with sample sizes greater than 100 (*n* = 6) were considered, the prevalence was 1% (95% CI 0–1%). However, when only retrospective studies with sample sizes less than 100 were included (*n* = 4), the prevalence was 29% (95% CI 6–100%); in prospective studies (*n* = 6), the combined prevalence was 11% (95% CI 4–29%). we performed a subgroup analysis of the differences in the incidence of IBD-BE between the different studies, each of which we subgrouped by type of study, type of disease, duration of disease, and diagnostic modality, and the results showed no significance. Future studies should standardize methods to identify IBD-BE cases and investigate the natural history and clinical course given the relatively high prevalence among IBD.

**Conclusion:**

In this systematic review and meta-analysis, the prevalence of IBD-BE was 12% among studies with HRCT imaging, suggesting that bronchiectasis may be an underestimated common extraintestinal manifestation of IBD. Asymptomatic patients with IBD-BE may present with abnormalities on HRCT or pulmonary function tests. Future studies should standardize methods to identify IBD-BE cases and investigate the natural history and clinical course given the relatively high prevalence among IBD.

## 1 Introduction

Inflammatory bowel disease (IBD) refers to a group of non-specific chronic inflammatory diseases of the gastrointestinal tract of unknown cause, including ulcerative colitis (UC) and Crohn disease (CD). The pathogenesis of inflammatory bowel disease (IBD) involves interactions between environmental, genetic, microbial, and immunomodulatory factors (1). Patients with IBD frequently present with one or more extraintestinal manifestations, including gangrenous pyoderma, erythema nodosum, arthritis, uveitis, and various types of lung disease (2). Results from Karmiris et al. (3) showed that women, smokers, and patients who had previously had an appendectomy or other significant surgery linked to IBD were more likely to have extraintestinal comorbidities. Kraft et al. (4) originally described pulmonary involvement in six IBD patients in 1976. Subsequent research has also demonstrated a high frequency of co-morbidities of asthma or COPD with IBD (5, 6). A large retrospective study in the United States indicated that the incidence of asthma among patients with inflammatory bowel disease (IBD) is 8.6%, other chronic obstructive pulmonary diseases is 5.9%, chronic bronchitis is 2.8%, pleural diseases is 1.9%, pulmonary fibrosis is 0.7%, pulmonary nodules is 0.7%, acute infectious bronchitis/bronchiolitis is 0.4%, and the incidence of bronchiectasis is only 0.3% (7).

There are multiple possible mechanisms for lung involvement in patients with IBD. Firstly, the lungs and the gastrointestinal tract share a common origin from the primitive foregut. Secondly, similar immune systems in the lung and intestinal mucosa (8), these could explain the occurrence of bronchial inflammatory changes in IBD patients. Vedolizumab is an intestinal-selective monoclonal antibody that has been found to be effective in the treatment of moderate-to-severe UC. It inhibits lymphocyte transport into the intestinal mucosa. In the study by Rizos et al. it was noted that a small number of UC patients developed interstitial lung disease shortly after treatment with vedolizumab (9), its implies a change in the target of the abnormal inflammatory response, which further supports the theory of “lung-gut axis” that the site of lymphocyte inflammation metastasis to the respiratory mucosa.

Patients with UC have been associated with a number of different patterns of lung involvement such as upper airway stenosis, tracheobronchitis, bronchiectasis, and interstitial lung disease (10). In addition to this, drug-related bronchopulmonary disease caused by the use of medication (sulfasalazine or mesalamine) has been reported in the UC patient population (11). However, reports of lung involvement in patients with CD are less common, but are associated with chronic bronchitis (12), bronchiectasis (13) and pulmonary infiltrates with peripheral blood eosinophilia (12). In this regard, high resolution CT (HRCT) of the chest can detect those abnormalities (14).

Both UC patients and CD patients can show lung involvement with bronchiectasis, but there are limited studies on the prevalence of bronchiectasis in patients with IBD, in which single-center, retrospective studies are common. And the reported prevalence of bronchiectasis in patients with IBD varies among studies, which may be related to the use of different imaging techniques to assess the lungs and the fact that some patients already had respiratory symptoms prior to inclusion in the study. As stated above, the prevalence of bronchiectasis is higher in IBD populations than in healthy populations (7), considering that the lungs and gastrointestinal tract share a common embryonic origin, and with the wide variation in the prevalence of bronchiectasis in IBD populations in the currently available studies, it is possible that bronchiectasis is an underestimated form of extra-intestinal involvement in IBD. The aim of this study was to investigate the prevalence of bronchiectasis in the IBD population, which could help to understand lung involvement in the IBD population as well as to determine the global prevalence of the disease, in addition, the risk factors associated with IBD-BE, which were enumerated in this study, may contribute to a better understanding of the pathogenesis and improved management.

## 2 Methods

We conducted a meta-analysis and systematic review of the prevalence of IBD-BE. We used the PRISMA-P 2020 list and pre-registered it (ID# CRD42024536409 on PROSPERO).

### 2.1 Search strategy and study selection

We queried PubMed and EMBASE databases. The search strategy was based on the PRISMA 2020 search strategy flowchart. We searched for “Inflammatory Bowel Disease/IBD/ Ulcerative Colitis/UC/ Crohn’s Disease/CD; AND; bronchiectasis.” No further restrictions were set to allow all possible results during this search. Inclusion and exclusion criteria were assessed by two independent abstractors for study eligibility. The search was conducted on 15 January 2024.

### 2.2 Eligibility criteria

The following specific inclusion criteria were applied in the screening process: peer-reviewed original science; reported at least 5 patients; published in English; and relevant to IBD and BE. To meet the prevalence target, we required the occurrence of IBD-BE to be identified in the larger denominator of patients with IBD. The following study characteristics were excluded during screening: non-primary literature (i.e., review articles, editorials); case reports or series involving fewer than 5 human patients; published in languages other than English; not related to IBD and BE; and studies not involving humans (e.g., mouse models).

We initially screened article titles and abstracts to remove articles that clearly did not meet the study criteria. After the initial screening, we conducted a full-text review to verify inclusion and exclusion eligibility criteria. All articles were independently reviewed by two individuals (YM and ZHQ) to validate inclusion and exclusion criteria. Data were independently extracted into a data extraction tool by two reviewers (YM and ZHQ). A third person (YL) adjudicated disagreements.

### 2.3 Summary measures

The primary purpose of this review was to summarize and report the prevalence of IBD-BE. The study population was defined as anyone with IBD according to the study criteria.BE was defined according to each study criterion and needed to occur in people with IBD. None of the included review studies included interventions due to a lack of interventional studies relating to this topic. All included studies were observational, which included cross-sectional studies as well as observational cohort studies, and the outcome of interest was IBD-BE. However, the length of follow-up in observational studies varied, which was mainly related to the study design.

Data measuring the prevalence of IBD-BE were extracted and validated. Relevant data to report the prevalence of BE in IBD included sample size, duration of IBD, number of BE cases in the IBD sample (molecular) and diagnostic methods, such as type and indication for chest imaging (clinical or research).

Quantitative meta-analysis of the prevalence of IBD-BE among IBD cases was performed using the R package. Considering clinical heterogeneity (e.g., inconsistent inclusion and exclusion criteria among studies) and methodological heterogeneity (e.g., differences in study design or blinding or duration of follow-up) in this study, and the meta-analysis results showed a high degree of statistical heterogeneity (>50%). Thus, we used a random-effects model for data analysis and sensitivity analyses as well as subgroup analyses as a means to analyze the sources of heterogeneity and their impact on the combined results.

We conducted a series of sensitivity analyses for the meta-analyses. Sensitivity analyses were performed using either a random-effects model or a fixed-effects model and considering joint retrospective and prospective studies, as applicable to the deleted studies. Secondly, we removed three large retrospective studies and one prospective study due to the lack of reference to the diagnosis of bronchiectasis (1, 3, 7, 15). Third, we excluded studies with fewer than 50 patients, including two retrospective studies and three prospective studies (10, 11, 13, 16, 17).

Fourth, we removed studies that used study chest radiographs rather than chest computed tomography (CT) (1, 3, 7, 15, 18).

Fifth, we limited our analyses to studies that used high-resolution (HRCT) scans as a chest imaging modality (10, 11, 16, 17, 19–22).

### 2.4 Risk of bias

To address risk of bias, two independent reviewers assessed the eligibility of the study and extracted data. A third reviewer completed the final assessment of the studies and data extraction to ensure that all included data were accurate. All studies were categorized according to the type of study design. We also quantified the type and presence of chest imaging. The study conducted a methodological quality assessment and provided a bias analysis (Table 1). The included studies were all of a retrospective design, which resulted in a higher risk of selection bias. The case series studies included exhibited significant heterogeneity. Other limitations include the small sample sizes of some of the included studies and the variability in imaging diagnostic methods. The results of this systematic review and meta-analysis must be interpreted with caution. In addition to this, we performed a publication bias test using the R package.

**Table 1:** Agency for healthcare research and quality (AHRQ) analysis and bias analysis results for studies included in the meta-analysis.

	P2022 (7)	S2022 (24)	K2016 (3)	H2017 (19)	G2018 (23)	M2018 (18)	G1993 (11)	S1997 (13)	S2011 (20)	A2023 (15)	M2000 (10)	D2011 (1)	J2012 (21)	N2002 (16)	Y2010 (17)	T2006 (22)
Define the source of information (survey, record review)	1	1	1	1	1	1	1	1	1	1	1	1	1	1	1	1
List inclusion and exclusion criteria for exposed and unexposed subjects (cases and controls) or refer to previous publications	1	1	1	1	1	0	1	1	1	1	1	1	1	1	1	1
Indicate time period used for identifying patients	1	1	1	1	1	1	1	0	0	1	1	1	0	1	1	0
Indicate whether or not subjects were consecutive if not population-based	1	1	1	1	1	0	0	0	0	0	1	0	0	1	1	1
Indicate if evaluators of subjective components of study were masked to other aspects of the status of the participants	0	0	0	0	0	0	0	0	0	0	0	0	0	0	0	0
Describe any assessments undertaken for quality assurance purposes (e.g., test/retest of primary outcome measurements)	1	0	0	1	0	0	1	0	1	0	1	0	0	1	0	1
Explain any patient exclusions from analysis	0	1	0	1	0	0	1	0	1	1	0	0	0	1	1	1
Describe how confounding was assessed and/or controlled.	1	0	0	0	0	0	0	0	0	0	0	1	0	0	0	0
If applicable, explain how missing data were handled in the analysis	0	0	0	0	0	0	0	0	0	0	0	0	0	1	0	0
Summarize patient response rates and completeness of data collection	0	1	1	1	0	1	0	1	0	0	0	0	1	1	0	0
Clarify what follow-up, if any, was expected and the percentage of patients for which incomplete data or follow-up was obtained	0	0	0	0	0	0	0	0	0	0	0	0	0	0	0	0
Count	6	6	5	7	4	3	5	3	4	4	5	4	3	8	5	5

## 3 Results

### 3.1 Study selection

**Figure 1:**
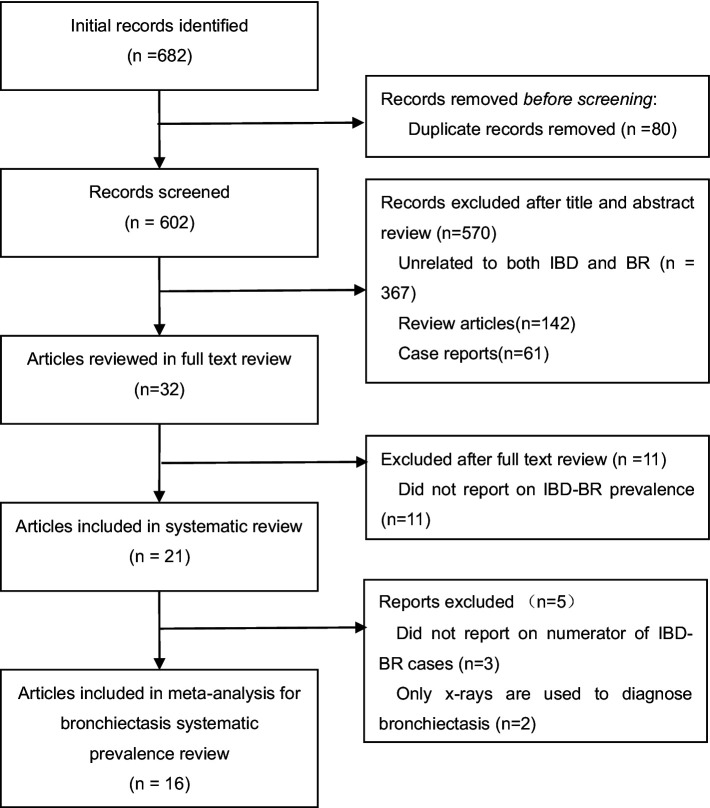
PRISMA flow diagram of studies assessed for bronchiectasis prevalence among patients with inflammatory bowel disease.

A total of 682 articles were identified through our database search. First, 80 duplicate articles were excluded. Second, in the initial screening of titles and abstracts, we excluded 142 review articles, 61 case reports, and 367 unrelated articles (see Figure 1 for flow chart). After screening full-text articles, we removed an additional 16 articles for the following reasons: prevalence of IBD-BE was not reported (*n* = 11); bronchiectasis was diagnosed using chest X-ray only (*n* = 3); and molecular IBD-BE was not mentioned (*n* = 2). In total, we included 16 full-text articles: 16 papers that all reported the prevalence of IBD-BE were included in the quantitative meta-analysis of IBD-BE prevalence.

### 3.2 IBD-BE in retrospective studies

Data related to prevalence collected in each retrospective study are shown in Table 2. Publication dates ranged from 1993 to 2023 and were conducted in the United States (*n* = 3), Japan (*n* = 3), Greece (*n* = 2), and Canada (*n* = 1). Mean IBD duration ranged from 2.7 to 19.85 years. In addition, five studies did not report IBD duration (7, 15, 18, 19, 23). The sample size ranged from 7 to 87,506 IBD patients. The diagnosis of bronchiectasis was not mentioned in 3 studies (3, 7, 15). Clinical CT or HRCT was used to diagnose BE in all but 1 study (11, 13, 18–20, 23, 24). Imaging modalities varied considerably from study to study, including blinding of clinical data, experience of radiologists, and number of radiologist reviewers.

**Table 2:** Retrospective studies reporting the prevalence of bronchiectasis among patients with inflammatory bowel disease. (*n* = 10).

Reference	Year	Country	IBD duration Years (unless specified)	Patients with Prevalent IBD-BR (numerator)	Total IBD Patients studied n (denominator)	Prevalence of IBD-BR in IBD (%)	Chest imaging modality used	Details on review of images and other notes
Pemmasani et al. (7)	2022	United States	Not reported	*n* = 267 (UC144 CD123)	*n* = 87,506 (UC 34229 CD 53077)	0.30%	Not reported	Not reported. IBD patients diagnosed by the International Classification of Diseases (9th Edition) Clinically Modified (ICD-9-CM)
Majima et al. (24)	2022	Japan	Mean 10.7 (SD 9.0)	*n* = 1 (CD 1 UC 0)	*n* = 136 (UC 76 CD 60)	0.73%	Research CT	Not reported. Patients with IBD were diagnosed according to the World Gastroenterological Organization Practice Guidelines for the Diagnosis and Management of IBD (2010), and all studies used thin-slice CT scans.
Karmiris et al. (3)	2016	Greece	Mean 2.7 (range 0.3–8.0)	*n* = 5 (UC 3 CD 2)	*n* = 1860 (UC 859 CD 1001)	0.26%	Not reported	Not reported. Patients were diagnosed with IBD at least 3 months prior to enrollment according to Lennard-Jones criteria
Sato et al. (19)	2017	Japan	Not reported	*n* = 5 (UC 4 CD 1)	*n* = 601 (UC 350 CD 251)	0.83%	Research HRCT	Not reported. Some patients have respiratory symptoms.
Grewal et al. (23)	2018	United States	Not reported	*n* = 9 (UC 0 CD 9)	*n* = 975 (UC 341 CD 634)	0.92%	Research CT	Not reported. All patients with IBD were diagnosed by pathological findings.
Mitsuhiro Moda et al. (18)	2018	Japan	Not reported	*n* = 14 (UC)	*n* = 660 (UC)	2.12%	Research X-Rays/CT	Not reported. Parts of patients have had colectomy and develop respiratory symptoms.
Garg et al. (11)	1993	United States	Mean 19.85	*n* = 7 (UC)	*n* = 7 (UC)	100%	Research CT/HRCT	Study was retrospectively reviewed by consensus of three radiologists. All patients had a history of cough and recurrent respiratory tract infections. Some patients do not develop respiratory symptoms until after total colectomy.
Spira et al. (13)	1997	Canada	Mean 12.2	*n* = 5 (UC 4 CD 1)	*n* = 7 (UC 6 CD 1)	71.42%	Research CT	Not reported. All patients had chronic sputum production.
Barge et al. (20)	2011	GETAID Centers	Mean 6.0	*n* = 25	*n* = 80 (UC 38 CD 42)	31.25%	Research HRCT	All data were reviewed and disaggregated by specialist panels of IBD and pulmonology (blinding unspecified).
Afroditi et al. (15)	2023	Greece	Not reported	*n* = 1 (UC 0 CD 1)	*n* = 70	1.42%	Not reported	Not reported. Some patients have respiratory symptoms.

### 3.3 IBD-BE in prospective studies

Prospective studies related to the prevalence of IBD-BE are shown in Table 3. The publication dates ranged from 2000 to 2012 and were conducted in countries such as Turkey (*n* = 3), the United Kingdom (*n* = 1), India (*n* = 1), and Poland (*n* = 1). Mean IBD duration ranged from less than 1 to 23.05 years. Sample sizes ranged from 17 to 95 patients with IBD. All studies used investigational chest HRCT for the diagnosis of BE, except for one study that did not mention the diagnostic method of bronchiectasis (1). Evaluation of imaging varied between studies, such as blinding of clinical data, experience of radiologists and number of radiologists evaluating, and was not reported in three studies (1, 21, 22). Two studies included only patients with IBD who did not have respiratory symptoms or airway abnormalities prior to enrolment (17, 22), and four studies included patients reporting respiratory symptoms or suspected lung abnormalities (1, 10, 16, 21). One study reported patients developing pulmonary symptoms after colectomy (10).

**Table 3:** Prospective studies reporting the prevalence of bronchiectasis among patients with inflammatory bowel disease (*n* = 6).

Reference	Year	Country	IBD duration Years (unless specified)	Patients with prevalent IBD-BR (numerator)	Total IBD Patients studied n(denominator)	Prevalence of IBD-BR in IBD (%)	Chest imaging modality used	Details on review of images and other notes
Mahadeva et al. (10)	2000	United Kingdom	Mean 23.05	*n* = 13 (UC 11 CD 2)	*n* = 17 (UC 14 CD 3)	76%	Research HRCT	Two radiologists were blind to the clinical history. Some patients develop pulmonary symptoms within 2 years of colectomy surgery.
Desai et al. (1)	2011	India	Mean 6.0 (range1-30)	*n* = 9	*n* = 95 (UC 83 CD 12)	9.47%	Not reported	Not reported. All patients diagnosed with IBD underwent inspiratory and respiratory HRCT.
Peradzynska et al. (21)	2012	Poland	Mean 23.2 months (SD 20.68 months)	*n* = 1	*n* = 50 (UC 25 CD 25)	2.00%	Research HRCT	Not reported. All patients underwent inspiratory and respiratory HRCT.
Songur et al. (16)	2002	Turkey	Mean 3.6 (SD 2.7)	*n* = 3	*n* = 36 (UC 23 CD 13)	8.33%	Research HRCT	Research physicians assessed patients imaging (blinding unspecified)
Yılmaz et al. (17)	2010	Turkey	Mean 3 (SD 0.5)	*n* = 2	*n* = 39 (UC 30 CD 9)	5.12%	Research HRCT	Research physicians blinded to clinical history. None of the patients had an occupational or family history of respiratory disease or atopy.
Tunc et al. (22)	2006	Turkey	Mean7.2(SD6.5) (range 0.1–35.0)	*n* = 5 (UC 3 CD 2)	*n* = 52 (UC 32 CD 20)	9.61%	Research HRCT	Not reported. All patients were included in the study with a questionnaire of respiratory symptoms, and patients with prior respiratory diseases were not included in the study.

### 3.4 Prevalence of IBD-BE among IBD meta-analysis results

**Figure 2:**
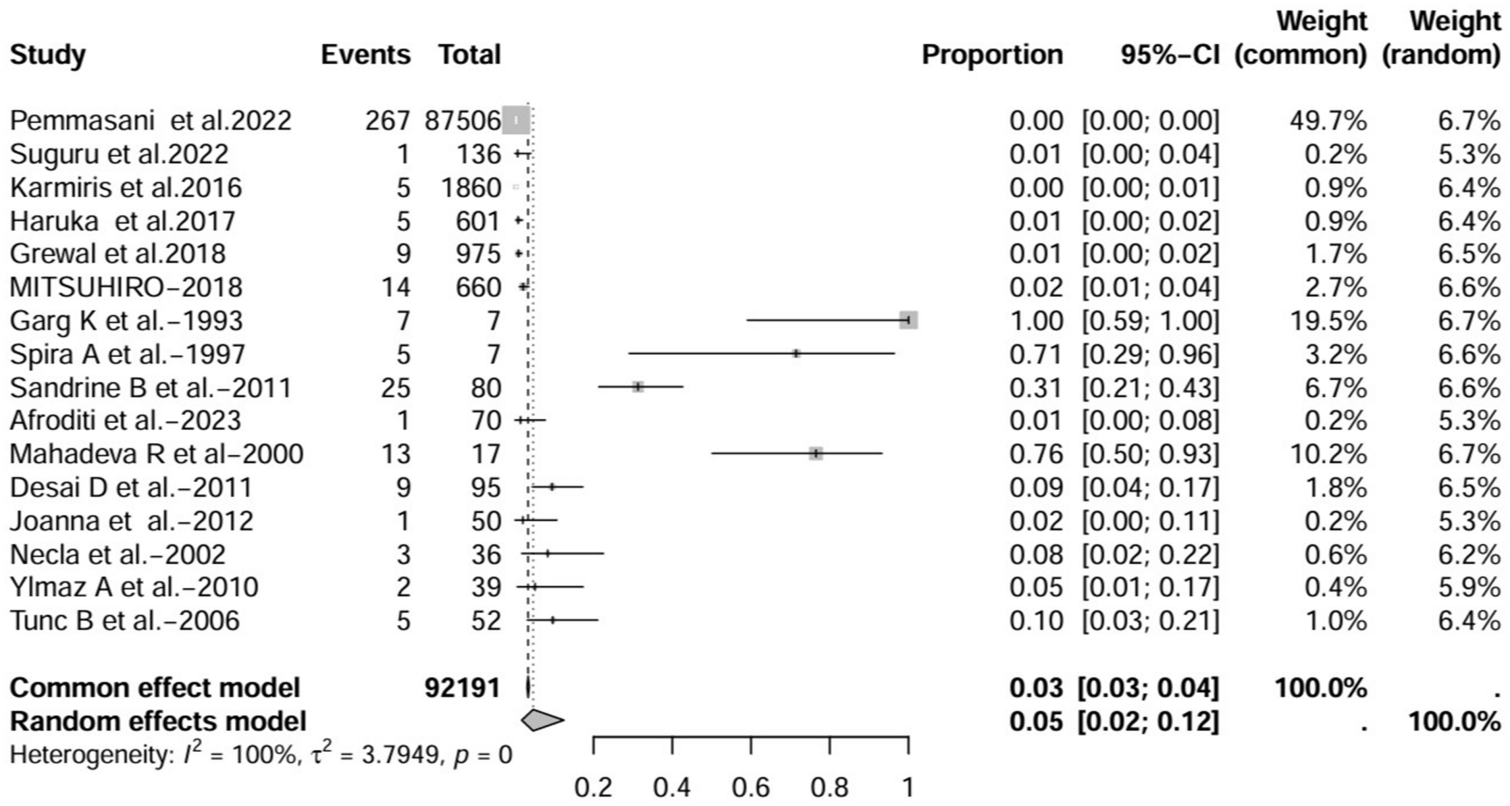
Forest plot and random effects meta-analysis of prevalence of bronchiectasis among patients with inflammatory bowel disease in all studies (*n* = 16).

**Figure 3:**
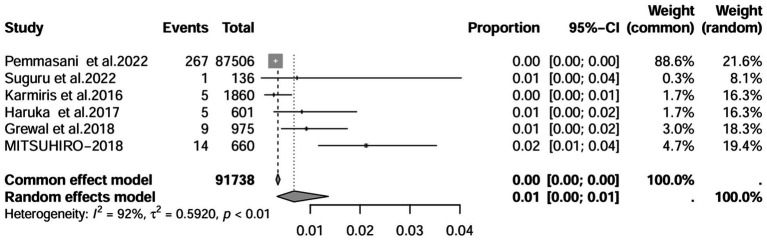
Forest plot and random effects meta-analysis of prevalence of bronchiectasis among patients with inflammatory bowel disease in retrospective studies with a sample size beyond 100 (*n* = 6).

**Figure 4:**
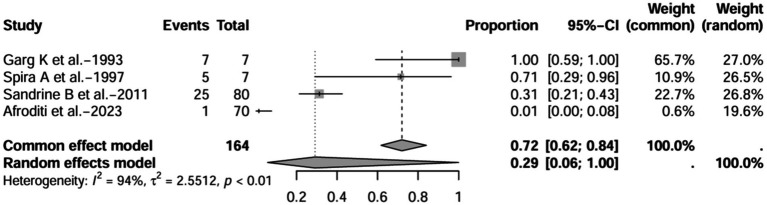
Forest plot and random effects meta-analysis of prevalence of bronchiectasis among patients with inflammatory bowel disease in retrospective studies with a sample size less than 100 (*n* = 4).

**Figure 5:**
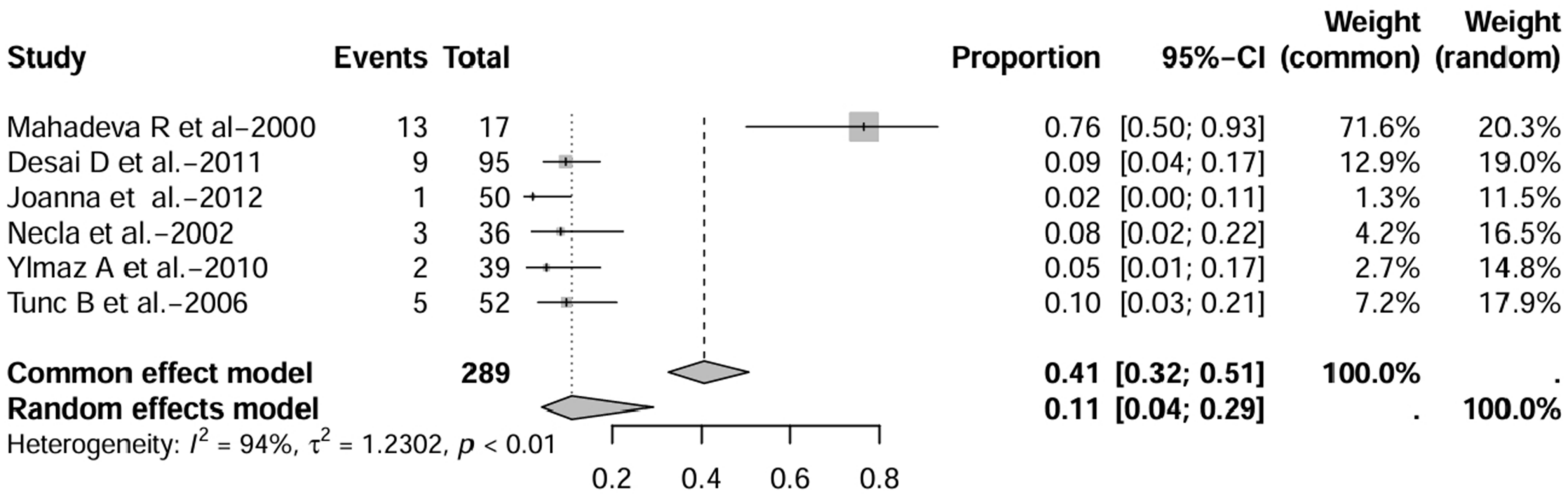
Forest plot and random effects meta-analysis of prevalence of bronchiectasis among patients with inflammatory bowel disease in prospective studies (*n* = 6).

A series of sensitivity analysis were performed to determine the prevalence of BE among patients with IBD reported in the literature. Forest plots for all studies are shown in Figure 2, sample sizes greater than 100 in retrospective studies are shown in Figure 3, sample sizes less than 100 in retrospective studies are shown in Figure 4, and prospective studies are shown in Figure 5.

In these 16 studies, 372 cases of IBD-BE were identified from a total of 92,191 IBD patients. In the preliminary analysis, the overall prevalence of IBD-BE in IBD in the random-effects meta-analysis was 5.0% (95% CI 2.0–12.0%; Figure 2). When only retrospective studies with sample sizes of more than 100 (*n* = 6) were considered, the prevalence of BE was 1% (95% CI 0–1%; Figure 4), however, when the sample sizes were less than 100 (*n* = 4), the prevalence of BE was 29% (95% CI 6–100%) (Figure 4); in prospective studies (*n* = 6), the prevalence of IBD-BE in the meta-analysis was 11% (95% CI 4–29%; Figure 5).

The lowest prevalence of IBD-BE has been identified in retrospective studies through clinical workups e.g., two large retrospective studies investigating 87,506 and 1,860 patients with IBD reported prevalence rates of IBD-BE of 0.30 and 0.26%, respectively (3, 7).

### 3.5 Sensitivity analysis

The sensitivity results of the meta-analyses are shown in Table 4. All analyses used random effects or common effects as the main analysis.

**Table 4:** Sensitivity results of meta-analyses.

Analysis combined	Number of studies included	Prevalence estimate	95% CI
Main analysis	16	0.05	[0.02; 0.12]
After excluding studies that did not report a diagnostic approach to bronchiectasis	12	0.08	[0.03; 0.22]
After excluding studies with fewer than 50 participants	11	0.02	[0.01; 0.05]
After excluding studies using x-rays to diagnose bronchiectasis	11	0.09	[0.03; 0.27]
Studies using HRCT for the diagnosis of bronchiectasis were included	8	0.12	[0.04; 0.39]
Retrospective			
In retrospective studies, in studies with a sample size greater than 100	6	0.01	[0.00; 0.01]
In retrospective studies, in studies with a sample size of less than 100	4	0.29	[0.06; 1.00]
After excluding studies that did not report a diagnostic approach to bronchiectasis	7	0.06	[0.01; 0.33]
After excluding studies with fewer than 50 participants	8	0.01	[0.00; 0.04]
After excluding studies using x-rays to diagnose bronchiectasis	6	0.33	[0.00; 0.67]
Studies using HRCT for the diagnosis of bronchiectasis were included	3	0.14	[0.01; 1.00]
Prospective			
Main analysis	6	0.11	[0.04; 0.29]
After excluding studies with fewer than 50 participants	3	0.07	[0.04; 0.11]
Studies using HRCT for the diagnosis of bronchiectasis were included	5	0.11	[0.03; 0.37]

**Figure 6:**
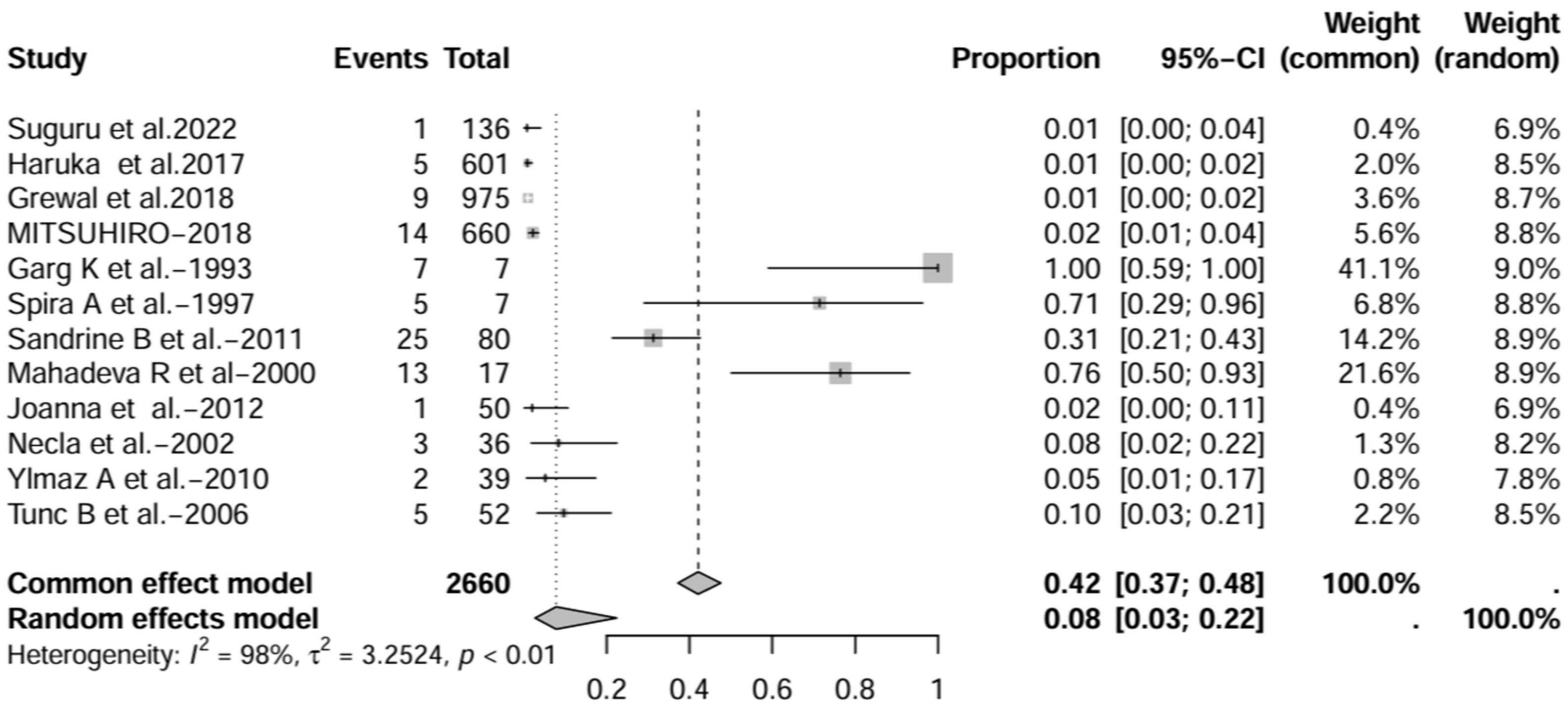
Forest plot and random effects meta-analysis of prevalence of bronchiectasis among patients with inflammatory bowel disease in all studies after excluding studies that did not report a diagnostic approach to bronchiectasis (*n*=12).

**Figure 7:**
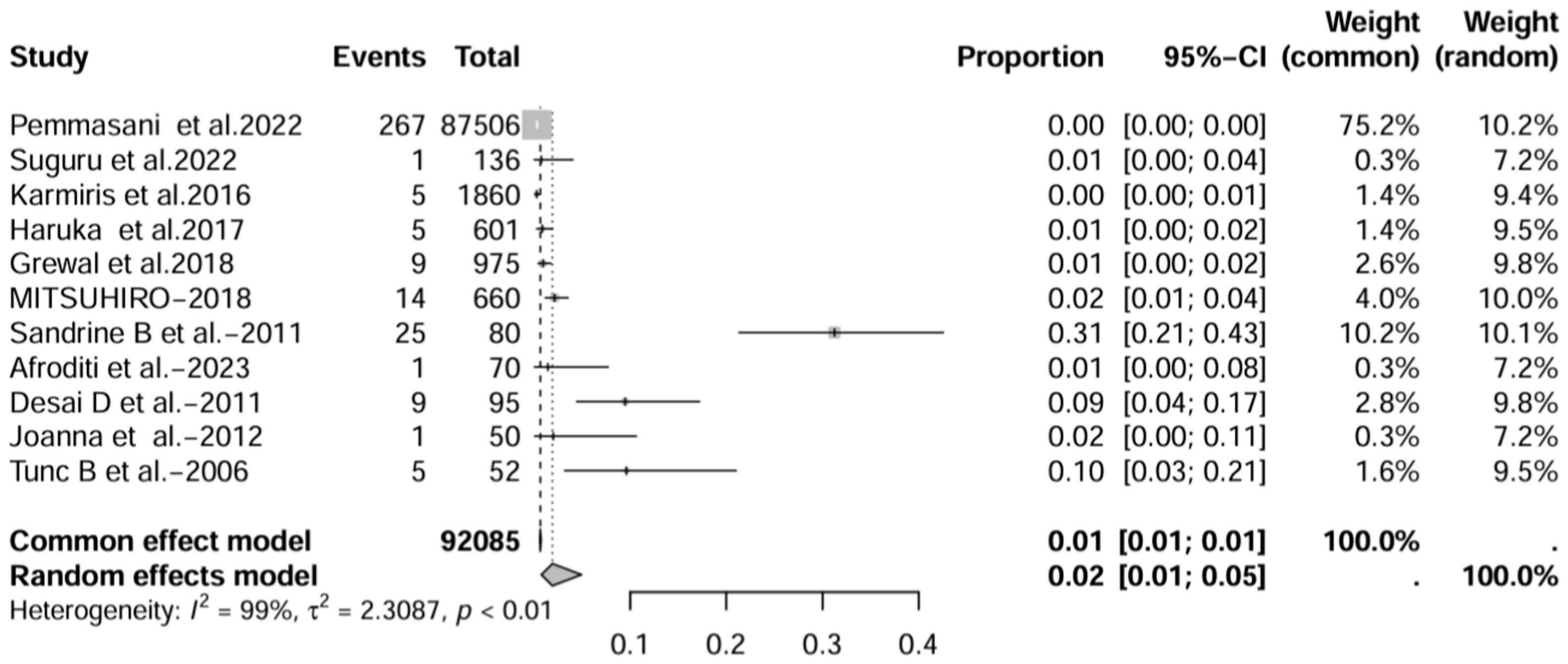
Forest plot and random effects meta-analysis of prevalence of bronchiectasis among patients with inflammatory bowel disease in all studies after excluding studies with fewer than 50 participants (*n*=11).

**Figure 8:**
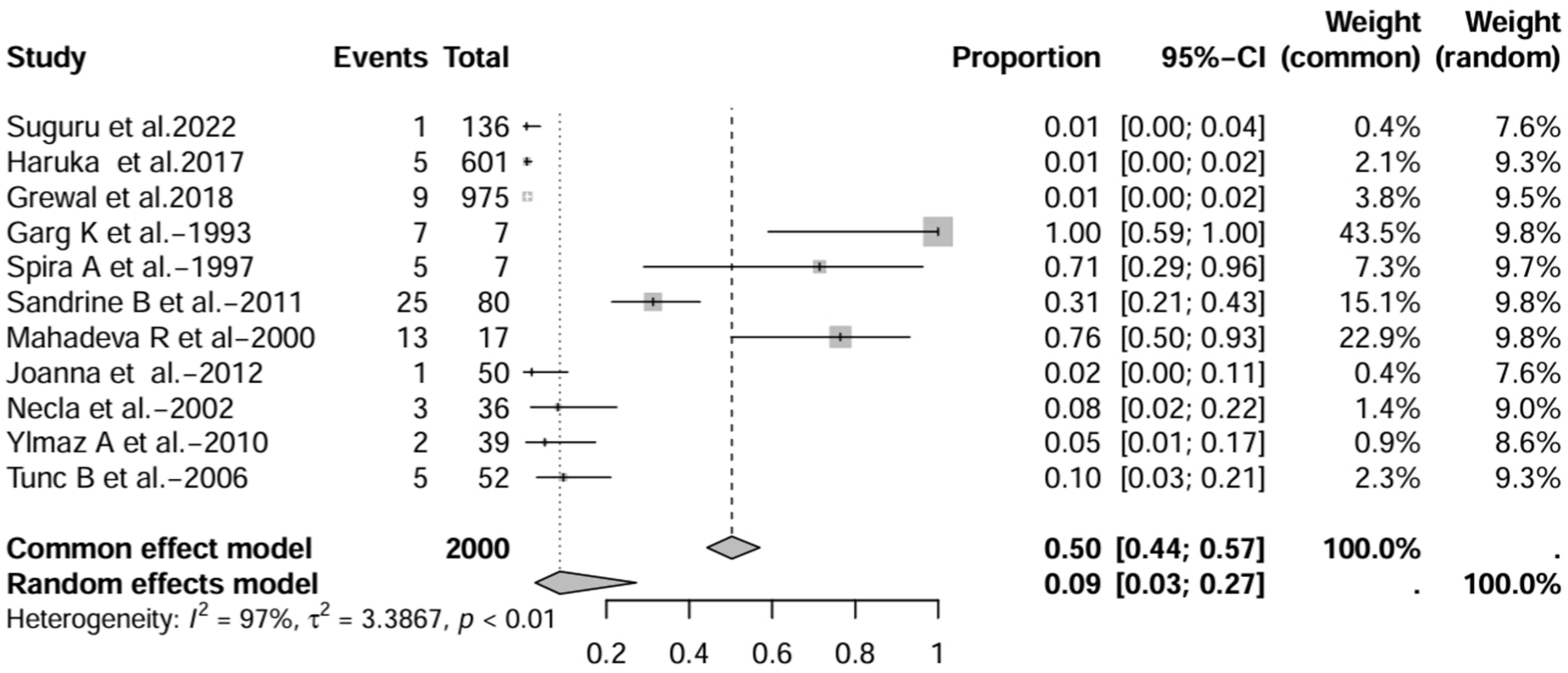
Forest plot and random effects meta-analysis of prevalence of bronchiectasis among patients with inflammatory bowel disease in all studies after excluding studies using x-rays to diagnose bronchiectasis (*n*=11).

**Figure 9:**
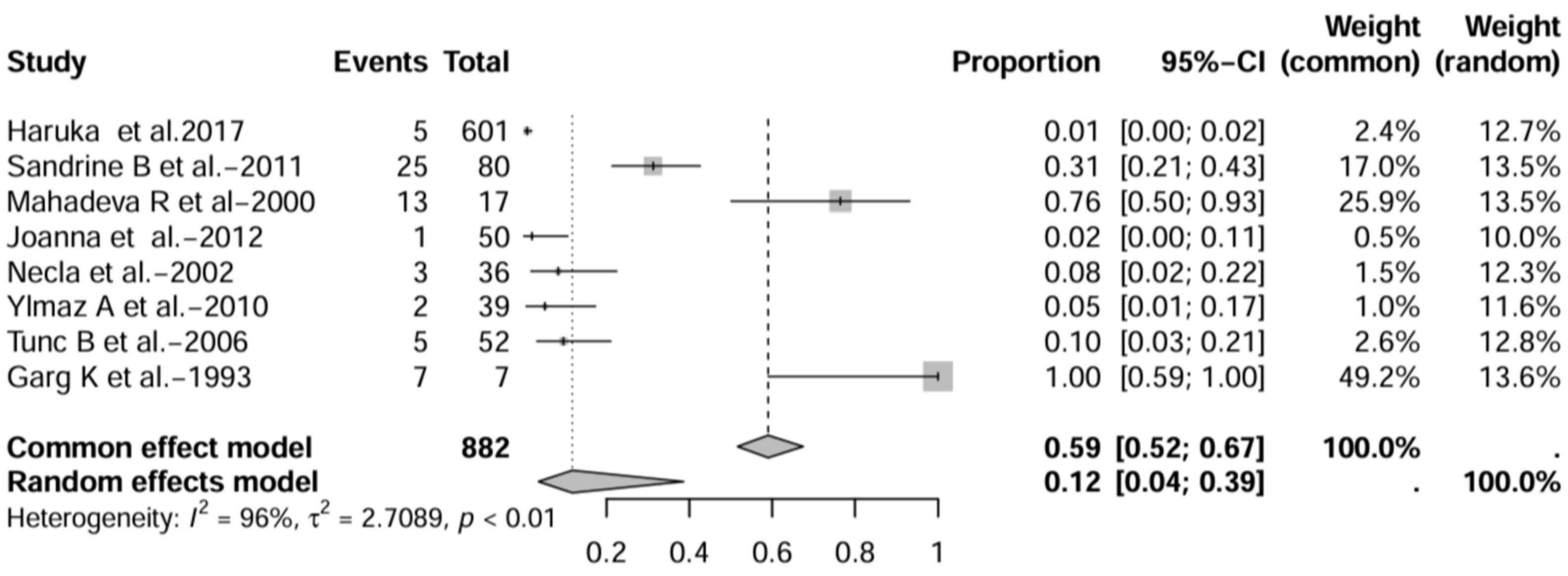
Forest plot and random effects meta-analysis of prevalence of bronchiectasis among patients with inflammatory bowel disease in all studies when included studies using HRCT for the diagnosis of bronchiectasis (*n*=8).

In all study (*n* = 16), after excluding studies that did not mention a diagnostic method for BE (1, 3, 7, 15), the prevalence was 8% (95% CI 3–22%; Figure 6). After excluding studies with fewer than 50 patients (10, 11, 13, 16, 17), BE prevalence was 2% (95% CI 1–5%; Figure 7). After removing studies that used radiographs to diagnose BE (1, 3, 7, 18), the prevalence was 9% (95% CI 3–27%; Figure 8). When we restricted the analysis to include studies with only HRCT scans (10, 11, 16, 17, 19–22) (*n* = 8), the prevalence was 12% (95% CI 4–39%; Figure 9).

Recommended diagnostic chest imaging modalities are critical for accurate diagnosis, and eight of these studies did not use HRCT scans (3, 7, 13, 15, 18, 23, 24). When these studies were excluded, the prevalence increased to 12.0%. Across all studies, there was significant heterogeneity in the methods used to interpret imaging scans and identify the population of patients with IBD-BE. This emphasizes the need for a reliable and consistent BE diagnostic method in patients with IBD.

**Figure 10:**
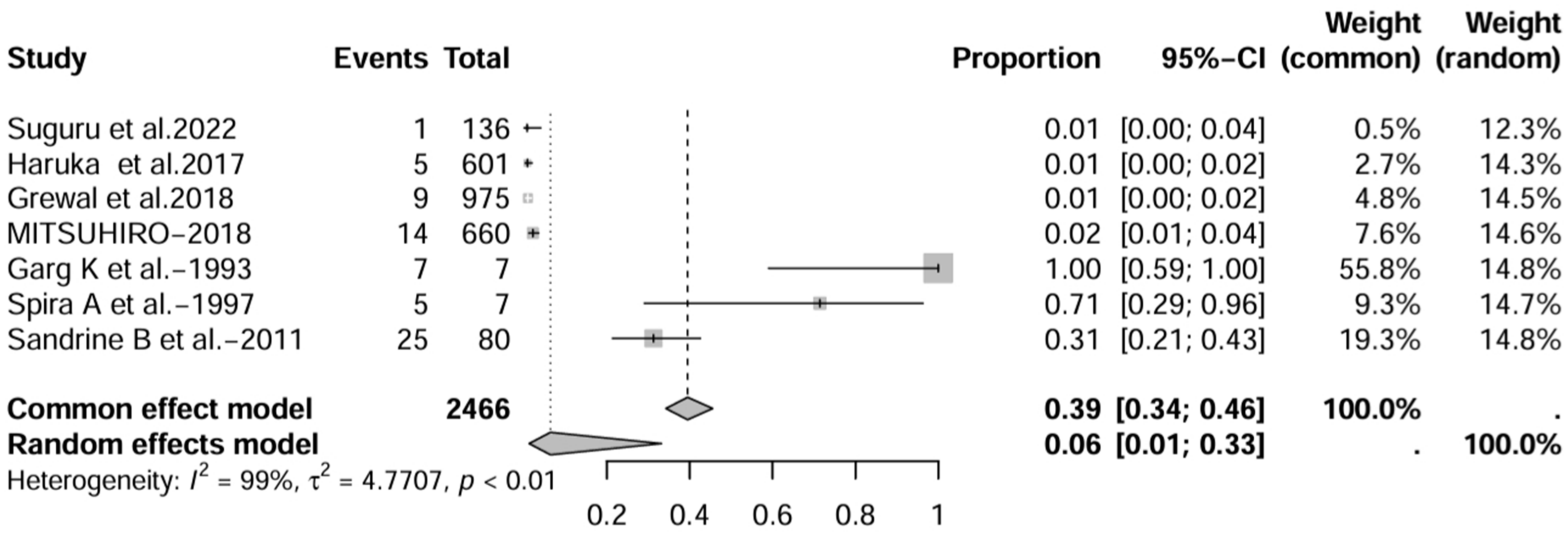
Forest plot and random effects meta-analysis of prevalence of bronchiectasis among patients with inflammatory bowel disease in retrospective studies after excluding studies that did not report a diagnostic approach to bronchiectasis (*n*=7).

**Figure 11:**
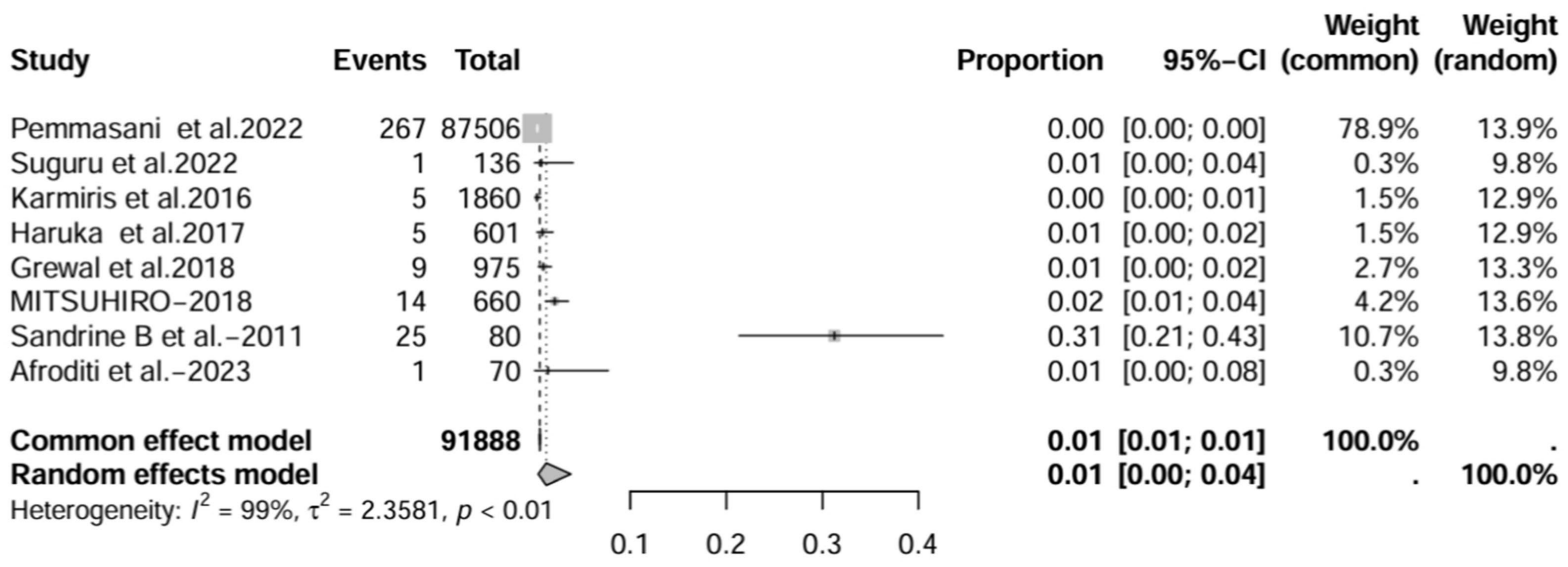
Forest plot and random effects meta-analysis of prevalence of bronchiectasis among patients with inflammatory bowel disease in retrospective studies after excluding studies with fewer than 50 participants (*n*=8).

**Figure 12:**
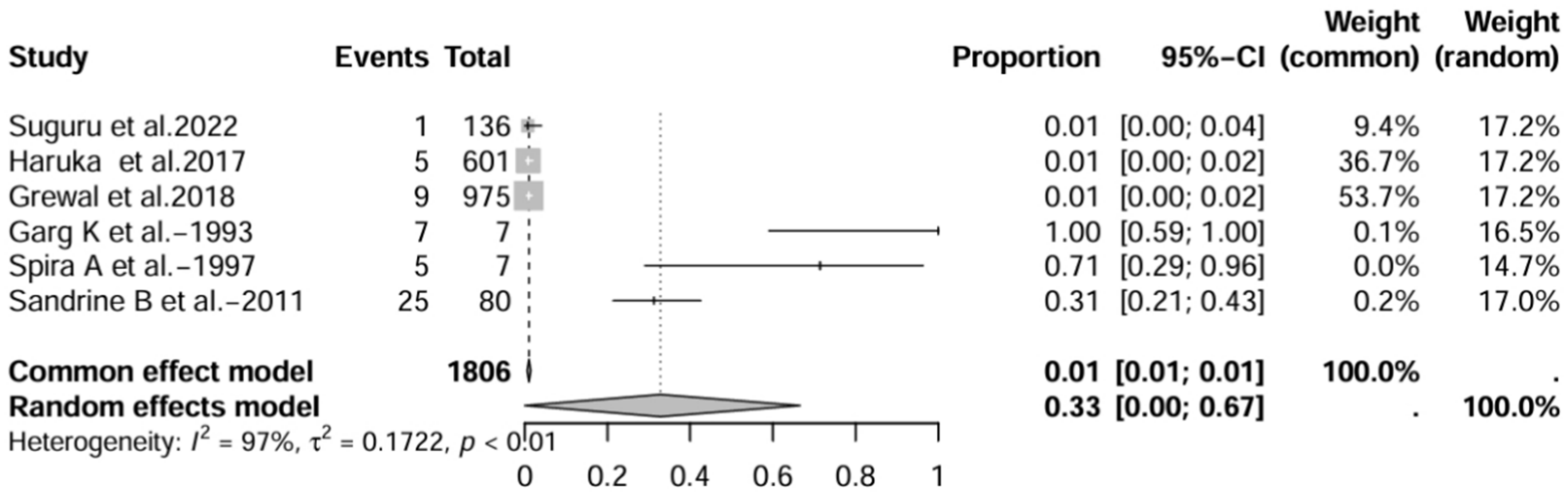
Forest plot and random effects meta-analysis of prevalence of bronchiectasis among patients with inflammatory bowel disease in retrospective studies after excluding studies using x-rays to diagnose bronchiectasis (*n*=6).

**Figure 13:**
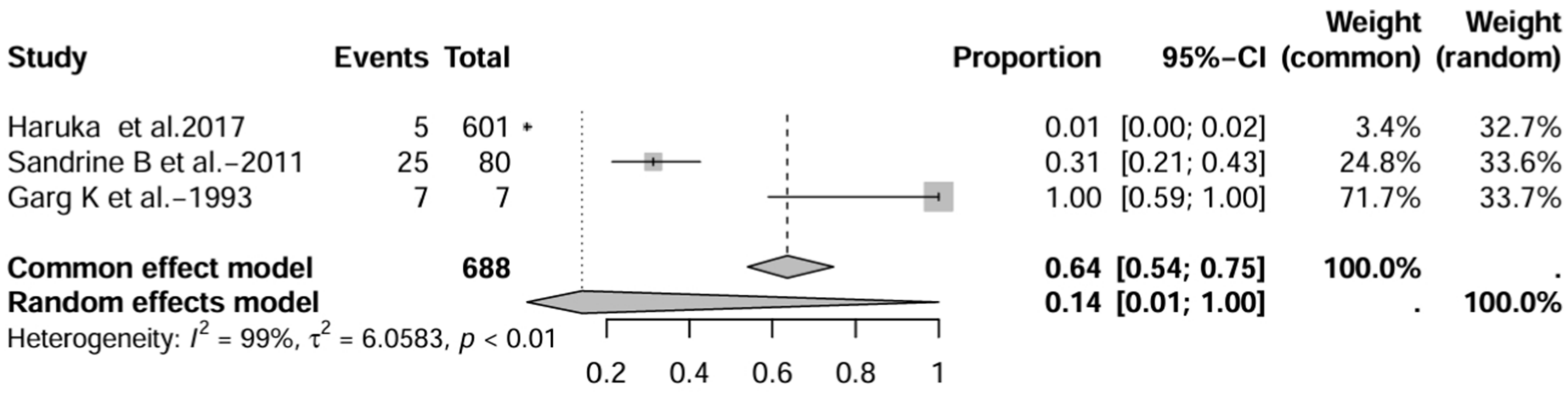
Forest plot and random effects meta-analysis of prevalence of bronchiectasis among patients with inflammatory bowel disease in retrospective studies when included studies using HRCT for the diagnosis of bronchiectasis (*n*=3).

In the retrospective study, the prevalence of IBD-BE was 6.0% (95% CI 1.0–33%; Figure 10) after excluding studies that did not mention the diagnostic method of bronchiectasis (3, 8, 15). After excluding studies with numbers less than 50 (25, 26), the prevalence was 1.0% (95% CI 0–4.0%) (Figure 11). After removing studies in which bronchiectasis was diagnosed using radiographs (18), the prevalence was 33.0% (95% CI 0–67%; Figure 12). Finally, after including studies that used HRCT to diagnose bronchiectasis (19, 20, 27), the prevalence was 14.0% (95% CI 1–100%; Figure 13).

In contrast to other studies, the prevalence of IBD-BE was reported to be lower than our pooled prevalence in some sample size studies (3, 7). Studies with large sample sizes and no mention of diagnostic methods for bronchiectasis may affect estimates of individual prevalence. For example, the 2 studies with larger sample sizes estimated the total number of IBD patients studied to be 87,506 and 1,860, respectively, but neither literature reported an imaging method for the diagnosis of bronchiectasis (3, 7), which could explain the prevalence rates of 0.30 and 0.26%, respectively, which were at odds with the prevalence rates reported by the studies using HRCT. As expected, the prevalence increased (to 8%) when we deleted all studies that did not mention a diagnostic method for bronchiectasis (1, 3, 7, 15). As mentioned earlier, most of the smaller studies had higher prevalence rates. After excluding 50 or fewer patients, the prevalence decreased to 2.0% (10, 11, 13, 16, 17), representing a significant portion of the overall prevalence studies in large studies. These results suggest that sample size and diagnostic imaging modalities have a significant impact on the prevalence of IBD-BE, and that longitudinal studies of large cohorts of patients with IBD are needed in the future, as well as the establishment of uniform diagnostic criteria for IBD and BE, in order to determine a more credible prevalence of IBD-BE.

These results suggest that sample size and diagnostic imaging modalities have a significant impact on the prevalence of IBD-BE, and that longitudinal studies of large cohorts of patients with IBD are needed in the future, as well as the establishment of uniform diagnostic criteria for IBD and BE, in order to determine a more credible prevalence of IBD-BE.

**Figure 14:**
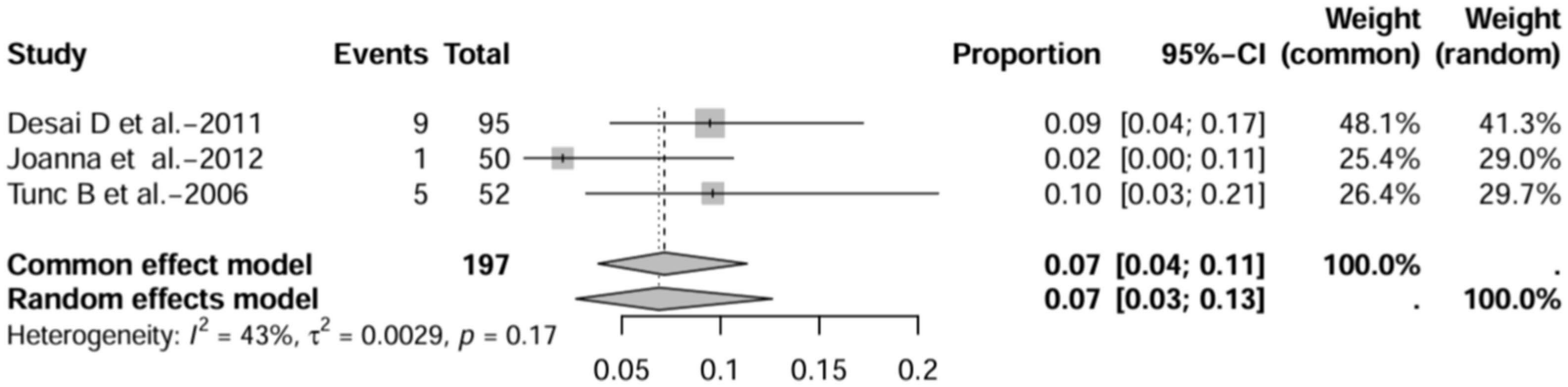
Forest plot and common effects meta-analysis of prevalence of bronchiectasis among patients with inflammatory bowel disease in prospective studies after excluding studies with fewer than 50 participants (*n*=3).

**Figure 15:**
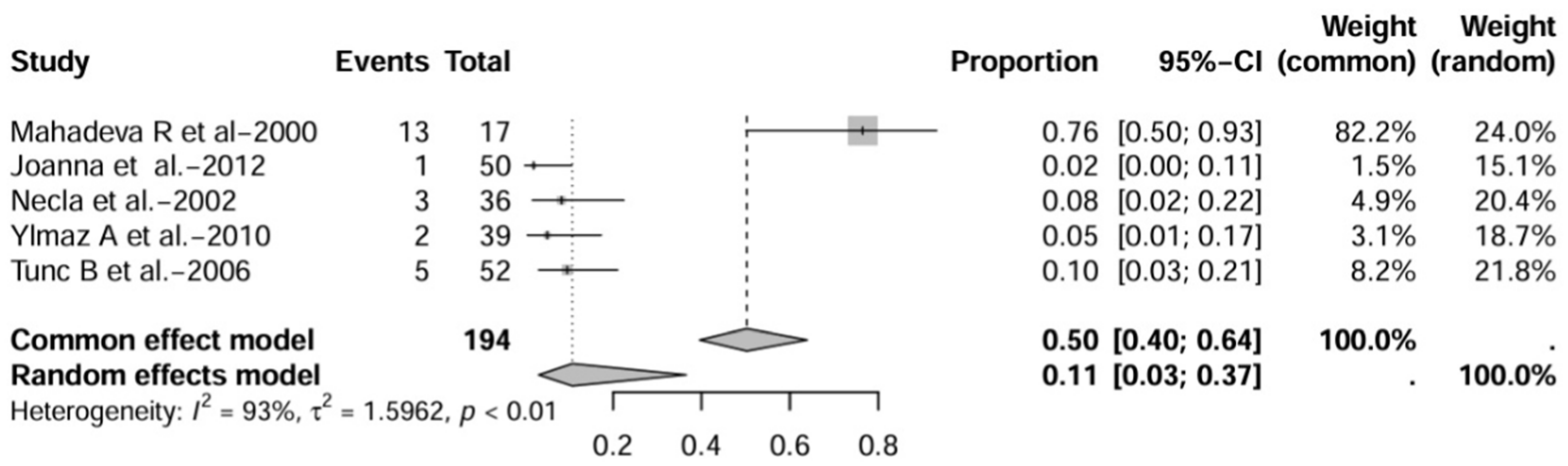
Forest plot and random effects meta-analysis of prevalence of bronchiectasis among patients with inflammatory bowel disease in prospective studies when included studies using HRCT for the diagnosis of bronchiectasis (*n*=5).

In prospective studies, the prevalence was 7.0% (95% CI 4.0–11.0%) after excluding studies with numbers less than 50 (12, 16, 17) (Figure 14). After inclusion of only studies that used HRCT to diagnose bronchiectasis (12, 16, 17, 21, 22), the prevalence was 11.0% (95% CI 3.0–37%; Figure 15).

In prospective studies, patients may be more willing to participate in the study if they had respiratory symptoms before enrolment, which may lead to selection bias. Some of these studies were designed to include patients with IBD (including those with ulcerative colitis and Crohn’s disease) to calculate prevalence and did not distinguish between disease types. In addition, the mean duration of IBD in prospective studies ranged from less than 1 year to 23.05 years. The differences in prevalence rates in the above prospective studies may be related to the differences in study design in terms of type of disease, duration of disease, and diagnostic method of BE (chest CT vs. HRCT vs. clinical symptoms) in the subjects. This implies that large prospective studies need to be conducted to determine the prevalence of BE in patients with IBD by examining and statistically analyzing patients with IBD by disease type, disease duration, diagnostic imaging methods for BE, population characteristics associated with respiratory symptoms, and pre-imaging screening for suspected lung involvement.

Whether retrospective or prospective studies, the sensitivity analyses described above suggest that improving HRCT in patients with IBD as much as possible can help to detect the onset of bronchiectasis at an early stage and help to guide the treatment of subsequent respiratory symptoms.

### 3.6 Subgroup analysis

**Figure 16:**
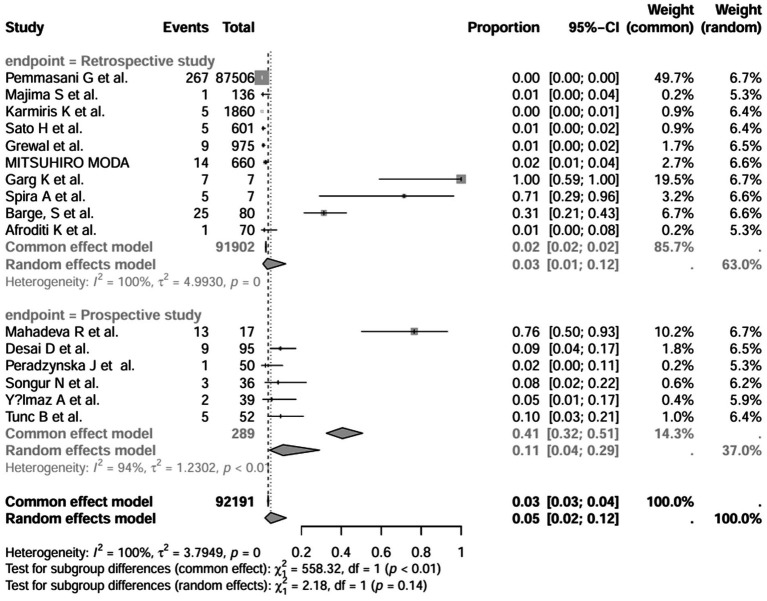
Subgroup analyses by study type grouping.

**Figure 17:**
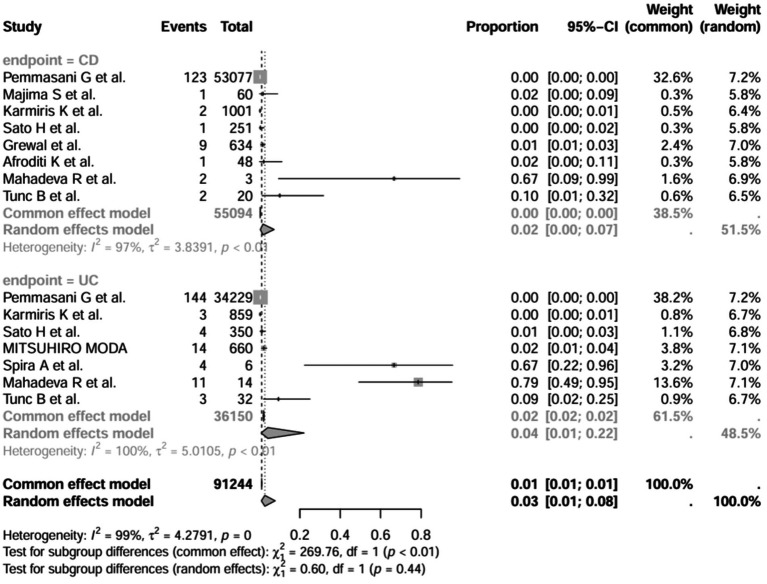
Subgroup analysis by disease type grouping.

**Figure 18:**
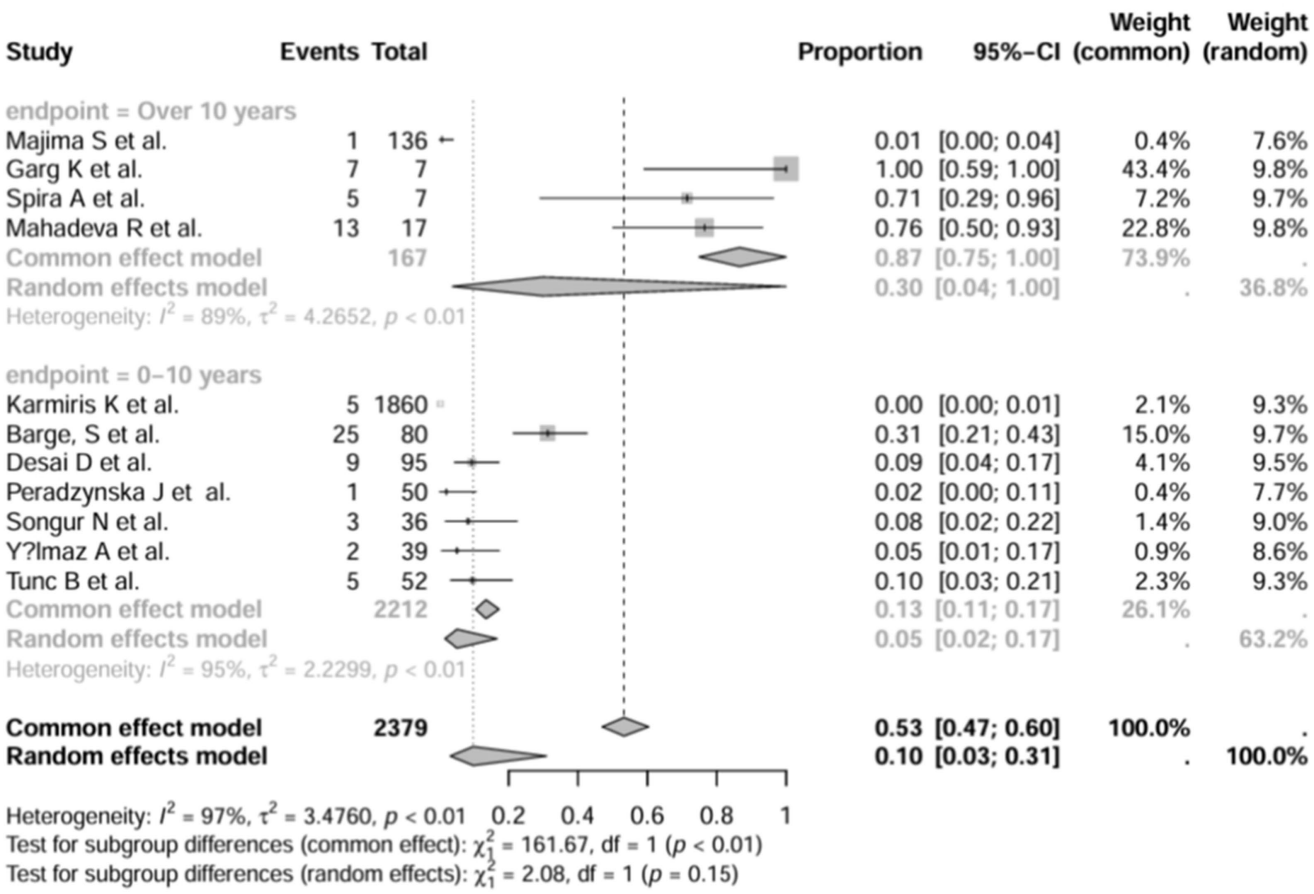
Subgroup analysis by disease stage.

**Figure 19:**
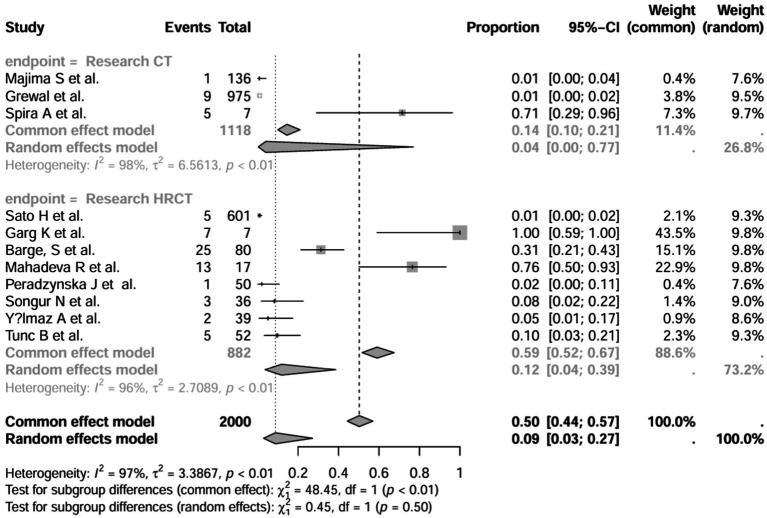
Subgroup analysis by diagnostic modality grouping.

In addition, we also performed subgroup analyses of differences in the incidence of IBD-BE between studies, and gender-related subgroup analyses were not performed in this study due to some missing information. The subgroup analyses of study type (Figure 16), disease type (Figure 17), disease duration (Figure 18), and diagnostic modality (Figure 19) were carried out in this study. It is worth noting that the results of the above subgroup analyses did not show significant differences, which suggests that multifactorial factors may play a role in the occurrence of bronchiectasis in patients with inflammatory bowel disease, and that large longitudinal cohorts of patients with inflammatory bowel disease are required to study the prevalence of bronchiectasis in the future.

## 4 Publication bias detections

### 4.1 Funnel plot

**Figure 20:**
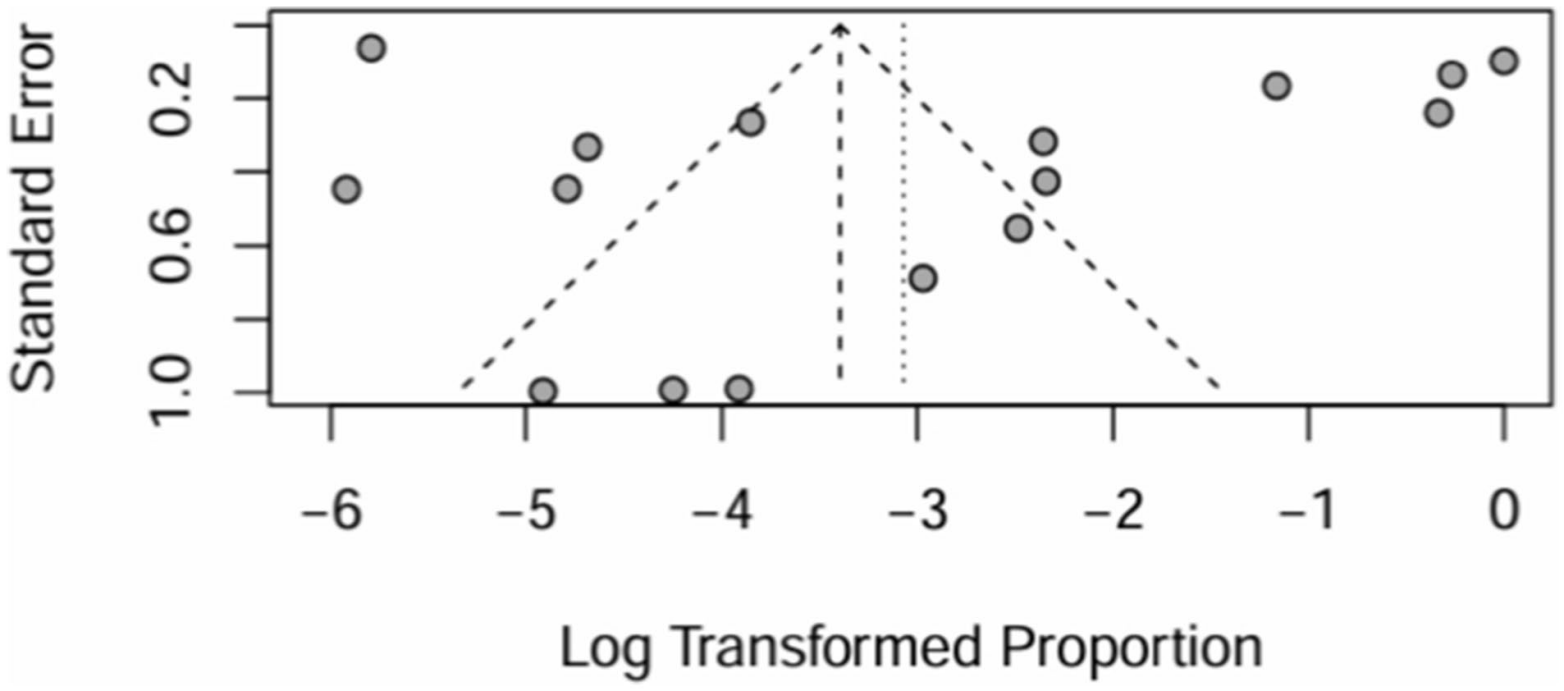
Funnel plot and random effects meta-analysis of prevalence of bronchiectasis among patients with inflammatory bowel disease in all studies (*n* = 16).

All 16 papers were included to draw a funnel plot, see Figure 20. The funnel plot showed a roughly symmetrical pattern.

### 4.2 Egger test

**Figure 21:**
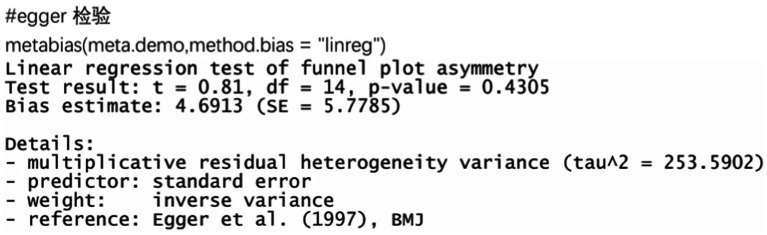
Egger’s test and random effects meta-analysis of prevalence of bronchiectasis among patients with inflammatory bowel disease in all studies (*n* = 16).

The funnel plots of all 16 papers included were plotted for the Egger test, see Figure 21. It can be seen that the *p* value = 0.4305 > 0.05, which is not significant, and there is no publication bias in the included papers.

## 5 Discussion

### 5.1 The pathogenesis of IBD

Inflammatory bowel disease (IBD) refers to a group of non-specific chronic inflammatory diseases of the gastrointestinal tract of unknown cause, including ulcerative colitis (UC) and Crohn disease (CD). The pathogenesis of inflammatory bowel disease (IBD) involves environmental factors, genetic composition, gut microbiota, and immune responses (28). Currently, the molecular mechanisms underlying intestinal inflammation are not fully understood. Some studies suggest that changes in gut microbiota, antibiotics, diet, smoking, and vitamin D may participate in immune regulation in the gut (29, 30). The pathogenesis of IBD is primarily associated with dysregulated immune responses in the intestinal mucosa, persistent intestinal inflammation, and increased permeability of the intestinal mucosal barrier. Dysregulated mucosal immune responses—characterized by pro-inflammatory cytokines released by CD4+ T cells and macrophages—are major drivers of barrier disruption (31). Furthermore, intestinal inflammation alters the gut microenvironment through high levels of pro-inflammatory cytokines (TNF-*α*, IL-1β, and IL-6), further activating the mucosal immune system (32, 33). The lung involvement in patients with IBD explored in this study may have multiple mechanisms of occurrence. Firstly, the lungs and the gastrointestinal tract share a common origin from the primitive foregut. Secondly, similar immune systems in the lung and intestinal mucosa (8), these could explain the occurrence of bronchial inflammatory changes in IBD patients.

### 5.2 The treatment of IBD

With a deeper understanding of the mucosal immune system and the pathogenesis of Inflammatory Bowel Disease (IBD), significant progress has been made in IBD treatment. The first-line treatment options for conventional IBD medications include aminosalicylates, immunosuppressants, and corticosteroids (34), aimed at reducing inflammation and alleviating moderate to severe levels of IBD inflammation (35). Additionally, inhibitors targeting the pro-inflammatory cytokine TNF-α can be used in the treatment of IBD patients, such as infliximab, adalimumab, and certolizumab pegol (36, 37); therapies with IL-10 and IL-11 can restore the balance between pro-inflammatory and anti-inflammatory cytokines; and anti-adhesion agents (such as natalizumab and vedolizumab) can prevent leukocyte infiltration into the endothelium (38) and block intracellular signaling (39). Some studies have also shown that prebiotic and probiotic interventions can help enhance the symbiotic gut microbiota (40). In clinical practice, antibiotics are commonly used alongside other medications as adjunct therapy for active IBD, to treat specific complications of Crohn’s Disease (CD), or to prevent disease recurrence after surgery (41). Analgesics, anticholinergics, and antidiarrheals should be personalized based on the patient’s symptoms and may be administered in addition to anti-inflammatory medications (42). However, immunosuppressants, corticosteroids, and most biologic agents listed above may pose a higher risk of opportunistic infections and immune-related side effects for patients. Furthermore, there remains a subset of patients in clinical practice who do not respond adequately or at all to medication treatments. Future therapeutic options for IBD patients could consider precision treatments targeting CD4+ T cell cytokines or blocking leukocyte adhesion.

### 5.3 The findings of meta-analysis

Our screening of the literature revealed heterogeneity in study design and study methodology related to the identification and case definition of IBD-BE. Key factors such as study design, design of included patients, sample size, diagnostic methods, and demographic or clinical characteristics varied considerably across studies. For example, prevalence rates ranged from 0.30 to 100.0% in all included studies, from 0.30 to 100.0% in retrospective studies, and from 2.0 to 76% in prospective studies. Therefore, we performed sensitivity analyses as well as subgroup analyses separately.

The results suggested a higher prevalence in prospective studies than in retrospective studies, a higher prevalence in small retrospective studies than in large retrospective studies, and a higher prevalence in studies using HRCT than in studies using plain CT or radiographs. Our findings were derived by performing additional sensitivity analyses.

In contrast, the prevalence of IBD-BE reported in several previous studies was less than 1% (3, 7, 19, 23, 24), our study of sensitivity results suggested an overall prevalence of 5%. This discrepancy may be attributed to the differences in sample size, imaging methods used to diagnose bronchiectasis, and ethnicity of the study population. In the present study, considering only large retrospective studies (those with a sample size of more than 100), the prevalence of BE was 1%. However, in small retrospective studies (when the sample size was less than 100), the prevalence of BE was 29%. The large difference in the prevalence of IBD-BE when the sample size was different suggests that the sample size influences the statistics of the prevalence of IBD-BE. This may be due to the fact that in small retrospective studies researchers are more inclined to include those with respiratory symptoms, whereas large retrospective studies have no such inclination. This leads to the possibility that small studies may overestimate the prevalence of IBD-BE. Conversely, in large studies, some patients did not present with respiratory symptoms, and radiologists may miss some subclinical patients. Therefore, the results of our prevalence study should be interpreted with caution and more work is needed to establish true clinical and subclinical prevalence.

In this systematic review and meta-analysis, the prevalence of BE in IBD was 5.0% in the 16 studies included, however, the prevalence of IBD-BE in studies using high-resolution chest computed tomography (HRCT) imaging was 12%, which is roughly the same as the results of the previous prospective studies (1, 16, 22), highlighting that BE may be an under-recognized extra-intestinal feature of IBD. For example, the 2 studies with larger sample sizes estimated the total number of IBD patients studied to be 87,506 and 1,860, respectively, but neither literature reported an imaging method for the diagnosis of bronchiectasis (3, 7), which could explain the prevalence rates of 0.30 and 0.26%, respectively, which were at odds with the prevalence rates reported by the studies using HRCT. As expected, the prevalence increased (to 8%) when we deleted all studies that did not mention a diagnostic method for bronchiectasis (1, 3, 7, 15). This suggests that the use of HRCT for the diagnosis of bronchiectasis increases the detection rate of IBD-BE, which is statistically demonstrated by an increased prevalence of IBD-BE. It is clear that early refinement of HRCT in the IBD patient population may allow for early screening and recognition of lung involvement in IBD patients.

A total of 16 studies involved countries as diverse as Japan, the United States, the United Kingdom, Canada, Greece, Poland, India, and Turkey, and ethnic differences in the study populations may have contributed to the differences in prevalence rates reported in previous literature.

Subgroup analyses were conducted separately for gender, type of study design, diagnostic imaging method, disease type, and disease duration, and there were no significant differences in the analyses of the groups, except for the gender group, which could not be analyzed due to some missing data information, which suggests that the prevalence of bronchiectasis in patients with inflammatory bowel disease is associated with a multifactorial involvement. It is unclear which factors may have influenced the results of the study, as different study design methods and other variables were factors that influenced the results.

And, in recent years, an increasing number of studies have reported lung involvement in patients with IBD. A large study based on a Canadian population found that both asthma and bronchiectasis were more prevalent in patients with IBD, with asthma being considered the most common extra-intestinal comorbidity in patients with IBD (25). Both patients with UC and patients with CD can present with lung involvement and bronchiectasis (10, 13), and with a 46% higher rate of bronchiectasis in IBD patients compared to a non-IBD cohort (7), IBD-BE may be of significance. But studies on the prevalence of bronchiectasis in patients with IBD are limited, with single-center retrospective studies being the most common studies. To further explore the factors and risk factors that may be involved in the development of IBD-BE, we reviewed the relevant literature in an attempt to identify clinical features that may be associated with IBD-BE.

Abnormalities in lung function have been found in cohorts of patients with IBD compared to healthy controls (17, 26), and Bonniere et al. found the presence of alveolar lymphocytosis on bronchoalveolar lavage in patients with asymptomatic Crohn’s disease (27), which may be associated with subclinical.

In addition, Camus et al. proposed the hypothesis of a “lung-bowel axis” (12), whereby there is an association between colectomy and the development of lung disease. The hypothesis of Camus et al. is supported by several studies in which respiratory symptoms in patients with IBD may appear months to years after colon resection (14, 16, 43), and by the independent association between colon resection and the development of airway disease in a series of studies by Moda (44) and others. Moreover, some patients have no respiratory symptoms and show only subclinical involvement with impaired lung function. All these reasons have led to the relationship between IBD and lung involvement not being well appreciated, leaving clinicians to underdiagnose lung involvement in patients with IBD.

In addition, it has been found that the different subtypes of IBD patients present different lung manifestations, with lung parenchymal involvement seen in UC, whereas airway involvement is mostly seen in Crohn’s disease (23), and that bronchodilatation and chimeric attenuation airway manifestations are more common in patients with CD compared to UC. Among others, Ellrichmann et al. (45) measured by FEV1/FVC ratio and they found a significant concordance between intestinal disease activity and airflow obstruction. In contrast, Kuzela et al. noted that there was no significant correlation between abnormal PFTs and bowel disease activity (46). The results of Tunc et al. also support the fact that there is no significant difference in lung function test results between patients with active disease in IBD and those in remission (22). The effect of disease activity on pulmonary function tests in patients with IBD remains a controversial topic.

Sato’s et al. concluded that there was no relationship between HRCT findings and disease activity (19), and Tunc et al. showed no correlation between HRCT abnormalities in IBD patients and IBD patients before the period of disease activity (22). Yilmaz et al. suggested that clinical activity in patients with UC was not associated with FEV1, FEV1/FVC, HRCT abnormalities, clinical/endoscopic disease activity, CRP, ESR or total IgE levels or body mass index (17). However, the study by Songu et al. states that approximately 80% of patients with pulmonary involvement have active bowel disease (16). Furthermore, the relationship between the duration and severity of IBD and the prevalence of lung involvement has also been reported in the literature (27, 47). These findings appear to be contradictory and do not lead to a consensus, and large longitudinal studies will be needed to confirm them in the future.

For child, the prevalence of bronchiectasis is lower in pediatric IBD patients compared to adult IBD patients, and it has been suggested that elevated levels of exhaled nitric oxide (FeNO) could be considered as a marker of airway involvement in children with non-smoking UC (21). Considering the paucity of studies in patients with IBD in childhood, more studies are needed to support this conclusion.

In this systematic review and meta-analysis, the prevalence of IBD-BE was 12% based on high-resolution chest computed tomography (HRCT) imaging studies, suggesting that bronchiectasis may be an underestimated common extraintestinal manifestation of IBD. The possible involvement of the lung-gut axis in the development of bronchiectasis in patients with IBD, including the common origin of the lungs and the gastrointestinal tract from the primitive foregut as well as the similarity of the immune systems of the lung and intestinal mucosa, could explain the development of bronchial inflammatory changes in patients with IBD. In addition to this, we performed a subgroup analysis of the differences in the incidence of IBD-BE in different studies, but the results showed no significant differences, suggesting that there is a multifactorial association with the development of bronchiectasis in IBD patients. Combined with previous reports in the relevant literature, this systematic review suggests that a number of factors, including the duration of inflammatory bowel disease, type of inflammatory bowel disease (UC versus CD), colon resection, disease activity scores, and subclinical infections, may be involved in the development of IBD-BE, which manifests itself in the form of new-onset respiratory symptoms, lung function, and HRCT abnormalities. As for pediatric IBD patients, elevated FeNO levels may be considered as a marker of airway involvement in non-smoking UC children. Here, the present study suggests that patients with IBD presenting with new, persistent and unexplained respiratory symptoms should be screened early for IBD comorbid subclinical lung involvement by tests such as HRCT and pulmonary function tests. Large longitudinal cohort studies of IBD-BE should be conducted in the future to determine the global prevalence of IBD-BE and to explore the underlying etiology and risk factors in patients with IBD-BE.

## 6 Strengths and weaknesses

The strengths of our systematic review and meta-analysis were the use of pre-specified inclusion and exclusion criteria and the extensive research and review of all relevant published articles on IBD and BE. Several sensitivity analyses were conducted to further support and clarify factors that may or may not have influenced the results. Limitations included the failure to clarify the potential etiology of bronchiectasis in the population of patients with IBD and the absence of studies on risk factors leading to bronchiectasis in the population of patients with IBD. Bronchiectasis may be isolated or secondary to structural distortion in fibrotic lung disease. Patients with isolated bronchiectasis may be more likely to require treatment directed at the primary airway dysfunction, including ciliary mucus clearance, antibiotics, bronchodilators, and azithromycin.

## 7 Conclusion

In this systematic review and meta-analysis, the prevalence of IBD-BE was 12% among studies with HRCT imaging, suggesting that bronchiectasis may be an underestimated common extraintestinal manifestation of IBD. Asymptomatic patients with IBD-BE may present with abnormalities on HRCT or pulmonary function tests. Future studies should standardize methods to identify IBD-BE cases and investigate the natural history and clinical course given the relatively high prevalence among IBD.
